# Vaccination into the Dermal Compartment: Techniques, Challenges, and Prospects

**DOI:** 10.3390/vaccines8030534

**Published:** 2020-09-16

**Authors:** Johanna Hettinga, Robert Carlisle

**Affiliations:** Department of Engineering Science, Institute of Biomedical Engineering, University of Oxford, Oxford OX3 7DQ, UK; johanna.hettinga@lincoln.ox.ac.uk

**Keywords:** vaccine, transcutaneous, intradermal, microneedles, ballistic delivery, DNA tattooing, skin permeabilization, electroporation

## Abstract

In 2019, an ‘influenza pandemic’ and ‘vaccine hesitancy’ were listed as two of the top 10 challenges to global health by the WHO. The skin is a unique vaccination site, due to its immune-rich milieu, which is evolutionarily primed to respond to challenge, and its ability to induce both humoral and cellular immunity. Vaccination into this dermal compartment offers a way of addressing both of the challenges presented by the WHO, as well as opening up avenues for novel vaccine formulation and dose-sparing strategies to enter the clinic. This review will provide an overview of the diverse range of vaccination techniques available to target the dermal compartment, as well as their current state, challenges, and prospects, and touch upon the formulations that have been developed to maximally benefit from these new techniques. These include needle and syringe techniques, microneedles, DNA tattooing, jet and ballistic delivery, and skin permeabilization techniques, including thermal ablation, chemical enhancers, ablation, electroporation, iontophoresis, and sonophoresis.

## 1. Introduction

In 2019, the WHO identified an ‘influenza pandemic’ and ‘vaccine hesitancy’ as two of the top ten challenges to global health [1]. A range of new vaccine technologies as well as processes to accelerate their development, testing, and implementation have emerged out of the remarkable global response to coronavirus disease (COVID-19). This impressive reaction will doubtless improve future readiness for, and response to, a wide range of potentially pandemic pathogens. However, amidst this fervent vaccine development activity, the second of those global challenges identified by the WHO, i.e., ‘vaccine hesitancy’, should not be forgotten. Improving the patient experience of vaccination will be an equally important part of ensuring effective preparation for, and reaction to, pathogenic disease. Especially, as many of the means of improving patient acceptance may also provide enhancements to both safety and efficacy compared to the current rather outdated paradigm of needle and syringe (N&S)-based vaccine delivery strategies.

In this review paper, the different routes of, and technologies for, vaccination will be explored, and the rationale behind developments in transcutaneous delivery and how these approaches are shaping the current vaccine landscape will be discussed.

## 2. The Skin as a Vaccination Target

The skin possesses multiple properties that make it an excellent site for vaccination: It is an easily accessible immune-rich environment and contains components that can induce both humoral and cellular immunity. It is also the natural route of infection for many pathogens so is evolutionarily primed to respond to challenge, an argument that may not hold true for the muscle.

**Figure 1:**
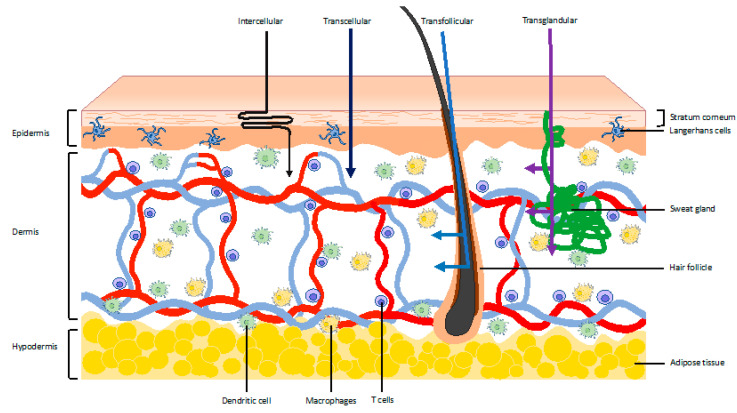
Schematic representation of the skin, the distribution of a variety of immune cells, and potential routes to bypass the stratum corneum.

The skin can be divided into three layers: The epidermis, dermis, and hypodermis (Figure 1). Vaccination to these layers are referred to as epidermal, intradermal, and subcutaneous, respectively, and distinct strategies exist for targeting the different layers [2].

The stratum corneum has a thickness averaging at around 20 µm, with the whole epidermis being around 85 µm thick, and it is the first and most formidable barrier against exposure to foreign substances and pathogens [3]. The stratum corneum is comprised of 10–20 layers of dead keratinocytes and intracellular lipids, proteins, and humectants [4,5]. Typically, lipophilic molecules with a molecular weight below 500 Dalton and which have less than three hydrogen bonding groups are able to pass through the stratum corneum [6]. Clearly, there are few vaccine strategies that match these specifications.

The stratum corneum can be split into four layers. From the outside to the dermis, these are the stratum corneum, stratum granulosum, stratum spinosum, and stratum basale.

As shown in Figure 1, there are four potential routes to bypass this layer: Intercellular, transcellular, transfollicular, and transglandular [7]. At the base of the stratum corneum are layers dominated by the keratinocytes, which will develop to form the stratum corneum. These keratinocytes form a crucial part of the innate immune system of the skin and express a variety of pattern recognition receptors and cytokines. Langerhans cells reside between the keratinocytes in the suprabasal layer and function as professional antigen-presenting cells (APCSs) [4,8]. Langerhans cells also possess the ability to sample the surface just below the stratum corneum by projecting dendrites through the keratinocyte junctions [9]. This has been shown to drive IgG1 responses in mice, activating humoral immunity against antigens [10].

The dermis is a fibroblast-rich environment with a network of elastin and collagen fibres to provide both strength and elasticity [11]. It is also interspersed with a network of lymph and blood vessels, as well as dendritic cells, macrophages, and T cells. Approximately 20 billion T cells, i.e., twice as many T-cells as in the entire blood volume, form perivascular layers throughout the entirety of the dermis [12]. Dendritic cells, however, are distributed interstitially near the epidermis and become progressively more numerous around the vasculature in the deeper layers of the dermis, similar to macrophages. The dendritic cells nearer the skin surface, which typically have a more branched morphology, form clusters and also express higher amounts of fascin, implying increased motility of these cells [12,13].

The hypodermis consists of large pockets of adipose tissue, which both insulates and provides protection, and a vertically orientated network of fibres, which form vacuoles. It functions as a reservoir for interstitial fluid, which can be used to change the stiffness of the tissue. The loose matrix structure of the hypodermis enables the free diffusion of inflammatory and cascading cytokines [14].

The heterogeneity of immune cells and their organisation in the different skin layers reveals how a variety of immune responses may be induced by targeting different subsets of the skin.

However, the composition and thickness of the skin is not a stationary state. It is impacted by age and BMI, shows great inter- and intrapersonal variability, and is interconnected with the human microbiome.

Knowledge on how skin thickness can differ is particularly relevant to ensure the desired subset of the skin is targeted. Measurements using ultrasound echography in Japanese children showed that the mean deltoid skin thickness was only 1.67 mm (1.16–2.39 mm) in 2-month-old infants and 1.81 mm (1.25–3.00 mm) in adolescents and a significant increase in thickness could be observed between 2 and 6 months for individual babies. For adolescents, a positive correlation was also observed between skin thickness and body weight [15]. A similar association was found for adults in a separate study [16]. There is also significant intrapersonal variation in skin thickness, with the waist having an average thickness of 1.55 mm compared to 2.54 mm in the suprascapular skin in adults of varying ethnicities. Women also tend to have slightly thinner skin (0.06–0.2 mm thinner) compared to their male counterparts. However, the skin thickness appears to remain relatively constant during adulthood (18–70 years), implying that needle lengths of approximately 1.5 mm will be broadly suitable for dermal targeting in the deltoid muscle region, waist, or suprascapular region for adults [17]. For infant vaccinations, on the other hand, adjustments in needle length or penetration depth may be necessary to account for their thinner skin. Ideally, devices that provide feedback on successful delivery into the dermal compartment would be used in the clinic.

The role the microbiome may play in modulating immune responses is a relatively new field. The skin is populated by a rich environment of viruses, bacteria, and eukaryotes, where the local bacterial population is heavily dependent on the physiology of distinct sites on the body (dry, moist, oily, follicle density) [18]. There has been speculation that a rich diverse skin microbiome is necessary for the education of distinct parts of the immune system, in line with what has been observed in the gut, and can alter immune responses [19]. For more details, De Jong et al. recently published a review on the impact of the microbiome on vaccine-induced immune responses [20].

## 3. Rationale for Vaccination into the Dermal Compartment

In the last few decades, in the race to develop novel vaccination technologies, there has been a renewed interest in the intradermal compartment as a target site, as it may enable dose sparing and broader immunogenicity compared to injections into the muscle. For instance, Arnou et al. demonstrated that intradermal injection of 9 µg of a trivalent inactivated split-virion influenza vaccine in humans achieved a similar immune response to intramuscular injection of 15 µg of the vaccine in adults aged 18 to 60 years [21]. In a study by Lang et al., rabies vaccines were shown to provide protective antibody titres in infants if a 0.1-mL dose of VERORAB™ was administered intradermally at 2, 3, and 4 months, compared with two injections of 0.5 mL of VERORAB™ at 2 and 4 months delivered intramuscularly, albeit with a lower total antibody titre observed in the intradermal group [22]. Although different dosages and administration regimes were compared in this study, an overall dose-sparing protocol was shown to be effective. However, it would be likely to increase the burden on healthcare staff. Intradermal vaccination has also been suggested as a dose-sparing strategy for the poliovirus vaccine, which has suffered issues with production and supply that are still being resolved [23,24]. Indeed, on the basis that equivalent or improved seroconversion rates were shown for two intradermal vaccinations using a fifth of the full 0.5-mL dose administered, compared to one full dose vaccine delivered intramuscularly, multiple countries, including India, Cuba, Bangladesh, and Ecuador, have adopted this strategy [25,26]. For influenza, a fifth of trivalent subunit influenza vaccines showed improved geometric mean titres and comparable seroconversion and seroprotection rates to full dose intramuscular injection in healthy young adults [27]. This is a result that agreed with a subsequent study in the elderly (≥65 years), who have shown higher seroconversion through intradermal injection of inactivated vaccine compared to intramuscular injection, which may suggest further advantages for intradermal vaccination for vulnerable groups [28]. A comprehensive review on intradermal vaccinations and their application in low- and middle-income countries has been published by PATH and WHO and complemented by a subsequent WHO bulletin [29,30].

As novel formulations of vaccines have been developed, transcutaneous and intradermal delivery has also been investigated as a method of increasing the potency of these new vaccines. For example, as interest in nucleic vaccines increases, because it offers a method of rapidly producing cheap versatile vaccines, different intradermal injection techniques are being looked at to improve immunogenicity [31].

Transcutaneous and intradermal procedures have been developed for both therapeutic vaccinations, which are primarily geared towards cancer vaccines and prophylactic vaccinations, which have centred around influenza vaccines but have also been investigated in the context of vaccines for hepatitis B, HIV, SARS-CoV-2, and dengue [32,33,34,35,36].

This review will address the advantages and disadvantages of targeting the dermal layers as a vaccination site and look at how different vaccination methods have developed to provide an effective means of delivery.

## 4. Vaccination into the Dermal Compartment by Needle and Syringe

Most people have experience with vaccines delivered using a needle and syringe (N&S) into the muscle. However, the first methods of providing protective immunity were via the skin through variolation, a practice that dates back to the early 16th century [37,38]. The history of inoculation has been well documented, resulting in much anecdotal evidence of the benefits of intradermal vaccination. The first references to deliberate intradermal vaccination can be traced back to Sutton and Dimsdale in the late 18th century, although no clear method is described [39]. The first formal method for intradermal injection was developed by Mantoux in 1910, when he developed a method to intradermally inject tuberculin to diagnose tuberculosis [2]. The Mantoux method has gone on to become the clinical standard for intradermal injection. The first study on the benefits of intradermal vaccine injection was reported in 1918 by Lieutenant L.T. Wright, who documented the advantages of intradermal vaccination against smallpox in the American Army compared to the traditional puncture, scarification, and incision methods [39].

These first studies on the benefits of intradermal injection were followed by multiple studies in the late 1920s and 1930s [40,41,42,43,44]. The often-cited study by Tuft reported a clinical trial comparing the intradermal injection of a typhoid vaccine with subcutaneous injection and reported an equivalent immune response with an improved adverse event profile for a lower dosage when injected intradermally [40]. This was followed by a range of clinical trials throughout the 1930s, including a study from 1936 when Thomas Francis and T.P. Magill intradermally injected medical students with influenza virus to raise protective antibodies and showed intradermal injection yielded comparable antibody titres to subcutaneous injection [45].

**Figure 2:**
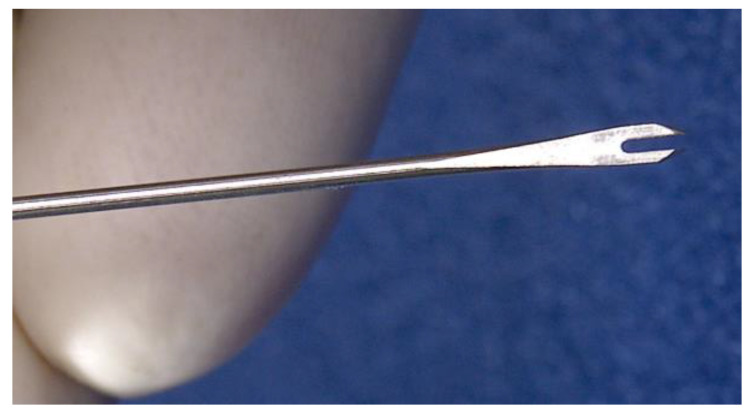
Bifurcated needle.

The development of the bifurcated needle by Benjamin Rubin, whilst working for Wyeth, LCC, was the next major innovation for intradermal injection. This device, as shown in Figure 2, picks up a consistent small volume of the vaccine, which can then be applied perpendicularly into the skin [46]. Even as recently as the 1970s, the smallpox vaccine was being administered intradermally using a bifurcated needle [47].

To increase reproducibility, more recently, a needle adapter for larger volume injections has been developed by PATH, which guides the angle and depth of the needle, mimicking the Mantoux method [48,49]. This is now commercially available through West Pharmaceutical Services, Inc. (West Whiteland Township, PA, USA) [50]. The first integrated microinjection system to reach the market was from Becton Dickinson (Franklin Lakes, NJ, USA), the BD Soluvia™ Micro Injection System [51]. This microinjection platform received marketing authorisation to use Soluvia™ for the intradermal influenza vaccine Intanza^®^, also known as IDflu^®^ [52]. Since 2011, Fluzone^®^ID has been approved by the FDA, which also uses the Soluvia™ system [32,53]. Fluzone^®^ID was withdrawn from the market after the 2017–2018 influenza season, and recent diminished use has probably been rationalised on a health economics basis.

The competing MicronJet600 device from NanoPass Technologies Ltd., Ness Ziona, Israel, has both FDA clearance and is CE marked for use in the EU. This device combines three pyramid-shaped microneedles and injects into the intradermal space, leading to the formation of a bleb [54]. Clinical trials (NCT01304563, IRB#1-2014-0026/study no.644, NCT01049490) have shown that it is effective for tuberculin skin testing and applying influenza vaccinations and in vitro work has shown promise for the delivery of plasmid DNA [55,56,57,58]. However, it failed to elicit comparable immune responses for a fractional dose of inactivated polio virus compared to intramuscularly injected vaccine [59].

More recently, VAX-ID^®^ was developed by Novosanis, Wijnegem, Belgium. It is a drug and vaccine delivery system that uses a 31G needle, which is perpendicularly injected into the dermal compartment. In clinical trials (NCT02186977, NCT01963338), the technology has been shown to be effective in hepatitis B booster vaccination and can show similar antibody titres as a 2.5x larger intramuscularly injected dose [60].

Similarly, Immucise^®^ by the Terumo Coorporation, Japan, has recently received FDA approval. The system utilises a 33G needle, which is 1.15 mm in length, and has a prefilled syringe that is placed perpendicular to the skin. In a phase I/II clinical study (JapicCTI-132096), the Immucise^®^ showed a similar safety profile to subcutaneous injection and a higher geometric mean titre for 15 µg of influenza haemagglutinin injected intradermally, with the novel device compared to subcutaneous delivery of an equal dose. It also suggested advantages for the use of the intradermal route in the elderly compared to subcutaneous delivery, as the seroprotection rate was about 70% for intradermal injection versus 48% in subcutaneous injection [61]. A follow-up phase III clinical trial (JapicCTI-142493) in the elderly (65 years and older) showed that 15 µg injected by Immucise^®^ provided higher geometric mean titres and seroprotection rates compared with 15 µg injected subcutaneously [62].

**Figure 3:**
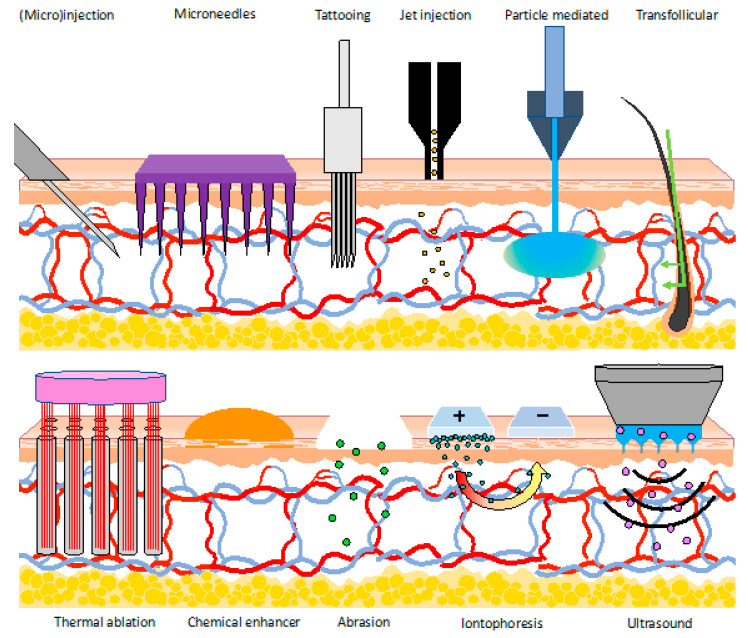
An overview of techniques used for vaccination into the dermal compartment.

Overall, these devices have shown good tolerability by patients and are most similar to currently used methods. However, it is also crucial to consider that the fear and pain relating to the use of N&S plays a big role in vaccine hesitancy, which is prevalent among patients, and so there is a strong desire to move away from these techniques [1,63]. Nevertheless, in moving away from transcutaneous/intradermal delivery on the basis of these human factor limitations, we need to ensure the baby is not thrown out with the bathwater. Indeed, there are a new raft of delivery technologies, which seek to evolve away from needle and syringe use but still retain the advantages provided by delivery into the skin (Figure 3).

## 5. Vaccination into the Dermal Compartment without Needle and Syringe

The ideal transcutaneous vaccine would provide protective immunity after a single dose and would be safe and cost-effective. The delivery method can significantly impact these factors, as well as affect patient compliance [64]. Considering pain is the biggest vaccine-related concern for parents and especially their children, alternatives to N&S, such as alternative pain-free transcutaneous methods of vaccination, offer the possibility of increasing vaccine compliancy, which as mentioned previously was identified as one of the 10 threats to global health in 2019 according to the WHO [1,65,66]. A recent meta-analysis showed that up to 90% of young children have a fear of needles and 16% of adults avoid influenza vaccines because of needle fear [67]. This needle avoidance also carries through to the parental vaccinations decisions and needle-sensitive parents are 9% more likely to delay HPV vaccinations for their children [68]. A situation where up to a fifth of the population may refuse the vaccine simply because of the method of its delivery makes a substantial negative impact on the already challenging targets required to achieve effective herd immunity for many pathogens.

The issues of the risks associated with cross-infection and hazardous waste resulting from needle and syringe use are also addressed by many of these alternative approaches. Indeed, a study in the Academic Medical Centre in Amsterdam, the Netherlands, showed that 66% of occupational exposures to potentially infectious material were attributed to needle stick injuries and a study in Germany showed that up to 37% of needle stick injuries involving a hepatitis B-positive donor resulted in seroconversion in a non-immunised recipient [69,70]. In total, it has been estimated that 16,000 infections of hepatitis C, 66,000 of hepatitis B, and about 1000 of HIV occur per year due to needle stick injuries in health care workers, which are unacceptably high numbers [71]. A review by Mannocci et al. also showed that significant costs are incurred in the management of needle stick injury, including lost productivity, laboratory testing, and potential treatments. These were estimated to equal $747 per needle stick injury in 2015 [72]. The risks of needle stick injuries do not only directly impact healthcare workers, but improper disposal also puts healthcare waste handlers at risk who may not have full knowledge of the risks associated with needle stick injuries [73].

It is no surprise therefore that a raft of new technologies has been developed, which aim to take advantage of the benefits of skin targeted vaccination without the downsides associated with N&S use. These technologies include microneedles and tattooing, jet and ballistic/particle-mediated delivery, and permeabilization approaches, which have relied upon thermal ablation, chemical enhancers, abrasion, iontophoresis, electroporation, or ultrasound [74,75,76,77,78,79,80,81,82,83,84,85]. This represents a huge variety of technologies, all applied with the common goal of circumventing the protective stratum corneum layer to access the immune rich milieu below (see Figure 1 and Figure 3). It is notable that, in addition to providing enhanced levels of safety and patient acceptance, these methods are also increasingly being utilised to achieve higher immunogenicity or improved delivery of a new range of vaccines, such as those based on DNA and RNA. The following section provides analysis of the benefits and limitations of these N&S alternatives in turn.

### 5.1. Injection Methods

#### 5.1.1. Microneedles

Topical and transdermal delivery of drugs has a long clinical history, with the first patch for transdermal delivery being approved in 1979, a scopolamine-delivering patch against motion sickness [80,84,86]. It was subsequently suggested that delivery could be improved by permeating the skin. This led to concept of microneedles around the 1970s, an approach that could not be investigated experimentally until the 1990s, when the microelectronics industry developed microfabrication tools for micron-sized structures [87]. Since their development, microneedles have been hailed as a pain-free alternative for transdermal drug and vaccine delivery and are currently under intense investigation. Approximately half the research around microneedle vaccines has been focused on influenza vaccines [88].

Microneedles (MNs) are arrays of micro-projections, which vary from 25 to 2000 µm in height [89]. The width of the needles ranges from 50–250 µm and the tips vary from 1–25 µm thick [90]. These micro-projections can have a range of geometries and create micropores that can be directly used to transport macromolecules, microparticles, or supramolecular complexes into the epidermis or the upper dermis [87,91]. Theoretically, superficially applied microneedles do not penetrate to the pain receptors within the skin, allowing for a pain-free delivery method. Hence, microneedles have the potential to allow for rapid onset of action, self-administration, and increased patient-compliance due to the simple and painless application mechanism [92]. This has been corroborated by volunteers in clinical trials, who reported little pain and viewed the possibility of self-administration as a great advantage. Specifically, in a structured questionnaire, 9 out of 10 volunteers rated the pain of application of an individual microneedle array between 0 and 3 on a scale of 0–10 and an equal number found it less painful than a hypodermic needle [93]. Further exploration of these aspects was performed by Norman et al., who showed that of the 44% who do get vaccinated for flu, 51% would prefer MN to N&S, and MN may be a means of persuading up to 30% of those who do not get vaccinated to do so [94]. However, a subsequent study reported 82% pruritus rates and 40% erythema rates for MN (vs. 16% and 0% for N&S) [95]. Hence, MN may alleviate rather than remove the immediate phobia and pain of N&S, whilst adding longer-lasting pain and irritation. Indeed, the issue of skin irritation and allergy, especially for microneedle tips, which break off the patch and remain in the skin, has been raised [90].

Notably, MN do not create hazardous waste and can allow for sustained release. However, the size of the MN is a limiting factor in the dosing, and typically needs to be less than ~10 mg and often less than 0.1 mg [88]. This has resulted in a particular interest in applying these microneedles to vaccination where such doses can still be efficacious if delivered effectively [88].

A variety of strategies have been devised to address the limitations of MN, including changing the materials used to form the needles. Currently, there are five main types of microneedles, solid, coated, hollow, dissolving, and hydrogel microneedles [90]. Within vaccination, the majority of research has been focused on dissolving microneedles.

##### Solid Microneedles

In their first iterations, microneedles were made from silicon [96]. Despite its flexibility in producing needles of varying size and shape, the cost and duration of production of silicon microneedles has seen limited application [90]. Solid microneedles are typically used as a pre-treatment, before the application of topical formulation in the form of an ointment or a patch [97]. This “poke and patch” method offers many advantages, such as sustained release and large doses, but can be more cumbersome for patients, as they need to wear the patches for extended periods [88]. Microneedle effectiveness as a pre-treatment for diphtheria toxoid vaccine was recently shown in mice by Ding et al. based on antibody titres. However, this result could not be recreated for influenza subunit vaccines, which was attributed to aggregates formed by the transmembrane domains of the haemagglutinin trimers and lower dosing [98]. This highlights how delivery and formulation must go hand in hand when developing effective vaccination strategies.

A variation on solid microneedles is nanoporous microneedles. These can deliver antigen based on diffusion from the microneedles into the skin and have elicited high levels of specific antibodies in an mouse model when exposed to the haemagglutinin protein and thereby provided complete survival after challenge with flu [99]. Trials reporting the impact of this technology in humans have yet to be reported.

##### Coated Microneedles

The next evolution was the production of coated MNs. One of the earliest applications of vaccine delivery with MNs involved dipping the microneedles in an ovalbumin (OVA) coating solution [100]. In the context of flu vaccine, solid microneedles coated with trivalent influenza vaccine tested in C57BL6 mice resulted in similar antibody levels to those achieved with an intramuscular injection using a ~100× greater dose (MN: 6.5 ng versus IM: 600 ng). Upon challenge, mice were completely protected from the mouse-adapted A/Puerto Rico 8/34 strain after single vaccination of 34 ng delivered by microneedles [101]. This impressive data further emphasizes the dose-sparing potential of the skin as a vaccination site. Plasmid DNA encoding hepatitis C virus NS3/4A protein coated onto stainless steel microneedles has also been able to elicit hepatitis C-specific cytotoxic C lymphocytes. Although no antibody titres were reported, vaccination with as little as 3.2 µg of plasmid using microneedles inhibited the growth of NS3/4A protein-expressing tumour cells, providing similar results to those achieved with 100 µg of plasmid injected intramuscularly [102].

Solid microneedles can also be used for micro-abrasions. These micro-abrasions have been shown to enable delivery of DNA by the “dip and scrape” method [87,103]. Considering the great interest in DNA and RNA as vaccine vectors, and multiple companies exploring DNA vaccines as a possible prophylactic treatment for coronavirus disease (COVID-19), the dip and scrape method could be a potential delivery route [104,105].

##### Hollow Microneedles

Hollow microneedles are most similar to traditional needles on a micro-scale and have a hole at the tip, which creates a channel for the passage of the vaccine into the dermis. It is particularly used for high-molecular-weight compounds, including proteins, vaccines, and oligonucleotides [90]. It has been suggested that antigen-loaded nanoparticles delivered by hollow microneedles yield a higher IgG2a antibody response and more interferon-γ-secreting lymphocytes compared to intramuscular injection. In support of this, Nui et al. demonstrated that OVA, imiquimod, and monophosphoryl lipid A encapsulated in poly(d,l-lactide-co-glycolide) (PLGA) nanoparticles resulted in higher anti-OVA IgG2a antibody titres and more interferon-secreting lymphocytes, compared to the same nanoparticles injected intramuscularly, in a Crl:CD(SD) rat model. However, this observation did not hold for soluble OVA delivered by a microneedle, which had similar IgG2a levels compared to intramuscularly injected OVA [106].

##### Dissolving Microneedles

For dissolving microneedles, the vaccine is incorporated in a sugar or polymer matrix and the needles are therefore designed to dissolve in the interstitial fluid. This does not create hazardous waste, which is an important advantage especially in enabling self-administration [88]. Dissolving MN have been shown to be stable for at least 6 months at 45 °C, allowing for the distribution of microneedles without the need for a cold-chain [107]. Dissolvable microneedles have been investigated for vaccination against a range of pathogens, including poliovirus, Shigellosis, influenza, hepatitis B, and SARS-Cov-2 [33,34,107,108,109,110]. Furthermore, they are capable of incorporating both antigens or nanoparticle-formulated nucleotides [107,111]. It is notable, however, that studies investigating nano-formulated DNA delivered into porcine skin with dissolvable microneedles did require the co-application of electroporation to elicit a strong immune response for a DNA porcine reproductive and respiratory syndrome virus vaccine. This also highlights the importance of human relevant models in preclinical work for translation to the clinic [112].

Recently, dissolvable microneedles have been tested for hepatitis B vaccination in rhesus macaques and were shown to elicit protective antibody levels and cellular responses. Specifically, after application of hepatitis B surface antigen-dissolving MN patches containing a 24 or a 48 µg dose for 20 min, 5 out of 7 animals had antibody titres above 10 mIU/mL, compared to 3 out of 4 after intramuscular injection of 10 µg of hepatitis B surface antigen. Microneedle vaccination also resulted in a higher induction of TNF-α levels, which has previously been shown to play a role in the migration of Langerhans cells to the lymph nodes [33,113]. In a separate study, dissolvable microneedles with hepatitis B surface antigen formulated with the adjuvant QS-21 also showed equivalent immunogenicity to intramuscularly injected hepatitis B with alum in pig models [107]. Other studies using pigs have also shown that dissolvable microneedles can be a feasible route of vaccination for hepatitis C [114].

Another interesting MN feature is that the time of dissolution can be altered to modify the type of immune response and multiple studies have tested different fabrication methods. Boopathy et al. described silk fibroin protein, which provides slowly dissolving microneedles on a quickly dissolving backing, the rationale being that once the backing has dissolved, the silk fibroin protein microneedles can remain in the skin to allow for sustained release. Its potential was demonstrated in BALB/c mice, where the novel microneedles were designed to release a cleaved stabilised soluble HIV envelope trimer immunogen and the adjuvants pam3CSK4 (a toll-like receptor (TLR) 2 agonist) and polyI:C (a TLR3 agonist) over a two-week period. This resulted in a 1300-fold increase in antibody titres compared to intradermal injection after one month and ELISpots showed that there was a significant increase in plasma cells and memory B cells after microneedle vaccination compared to a bolus intradermal injection. Analysis of follicular helper T cells showed that the number of these cells after bolus injection peaked after 7 days, whilst similar levels were reached by microneedles after 14 days [79]. Biodegradable microneedles formed with gelatin methacryolyl have also shown potential for the sustained release of chemotherapeutics in vitro [115]. This method of sustained release may also be applied in vaccination. Another method to increase the dissolution rate is increasing the antigen loading in the microneedles [116].

A clinical trial (NCT02438423) utilising a sucrose and gelatine-based dissolvable MN patch with an inactivated influenza vaccine showed antibody titres at day 28 that were comparable to an intramuscular injection control group as well as a similar seroconversion rate. It made no difference if the MN patch was applied by a healthcare worker or self-administered, showcasing the potential for self-vaccination with microneedles [95].

Dissolvable MN can likewise incorporate adenovirus vaccines, which can elicit cellular and humoral adaptive immune responses in naïve mice [117]. In future, adenovirus-based vaccines may be delivered by MNs as well.

As adjuvants play an important role in improving the immunogenicity, adjuvants have been incorporated directly into the microneedle, such as Poly(I:C), gibbsite, and toll-like receptor 3 (TLR3)-triggering double stranded RNA [117,118,119]. Although, it should be noted reports have varied on the efficacy of incorporating adjuvants into the microneedles [119]. The potential of using the dissolvable microneedles themselves as an adjuvant by manufacturing them from chitosan has yielded promising pre-clinical results in mice vaccinated with inactivated influenza vaccine formulated with implantable chitosan microneedles. Complete protection against 2 × 50% lethal dose of influenza challenge was observed compared to 40% survival for intramuscularly vaccinated mice. Additionally, higher antibody titres were observed compared to intramuscular vaccination of an equal dose of antigen, but the impact on the cellular response was not probed [110]. Information on the amount of skin area coverage used to achieve these results would be valuable in assessing scalability to human use.

The potential for the quick development of novel microneedle-based vaccination was shown in response to the COVID-19 crisis. Kim et al. rapidly developed a dissolvable microneedle array that incorporated the SARS-CoV-2-S1 or SARS-CoV-2-S1fRS09 protein in a carboxymethyl cellulose matrix. This resulted in potent antigen-specific antibodies just 2 weeks after administration of a prime vaccination and a boost after a further two weeks increased antibody titres even further in BALB/c mice. Neutralisation assays were not performed, as they were not available at that time for SARS-Cov-2 [34].

##### Hydrogel Microneedles

Hydrogel microneedles are made from cross-linked polymers capable of swelling as a result of the uptake of interstitial fluid. These filled microneedles form conduits that allow continuous patch-type drug reservoirs to release compounds for sustained release [120]. Unfortunately, some early mouse studies that compared vaccine administration of hydrogel microneedles with dissolving microneedles failed to achieve similar IgG titres to dissolving microneedles [121].

Other developments in microneedles have included thermoresponsive microneedles. These allow for release when the microneedles are heated above a threshold or for the viscosity of the microneedles to be tuned through temperature, allowing for tweaking of the release profiles [122,123].

In summary, a great range of microneedles with a wide variety of properties have been created and shown potential in pre-clinical studies. To date, no microneedle array patches have marketing approval for vaccine delivery, although a dissolving microneedle patch with hyaluronic acid is marketed for cosmetic purposes, MicroHyala^®^ (CosMED Pharmaceutical Co., Ltd., Kyoto, Japan), and may help normalise the concept of microneedles [90]. There has been a marked lack of these technologies in clinical trials, despite the high hopes for this potentially pain-free delivery technique. Taking the step into clinical vaccine trials will require more research into reproducibility, greater confidence in dosing, and better control over the effect of different skin compositions. Furthermore, more research into the optimal parameters for vaccination, including adjuvants, release profiles, and stability, is needed. Ultimately, all these microneedle systems provide a means of providing a localised increase in the concentration of vaccine, and so the process of dosing is still both driven by and limited by diffusion. Currently, the large numbers of trials needed for approval, limited dosing of current patches, and common adverse effects, such as allergies, redness, and irritation, have prevented this technology from fulfilling its promises and entering the market [90]. Its nature as a combination product, requiring the development of both a suitable microneedle formulation and compatible active compound, may also have hampered its industrial development, something that is further complicated by the need to develop novel industrial production processes that ensure uniform, large-scale, and sterile production of microneedles. Particularly, evaluating uniformity and developing sterilisation methods that do not compromise the stability of microneedles has proven difficult and prevented marketing approval [124]. If these hurdles can be overcome, microneedle technology can start fulfilling its potential.

#### 5.1.2. DNA Tattooing

DNA tattooing involves puncturing the skin thousands of times with a multiple needle tattoo device or permanent make-up device to deliver plasmid DNA vaccine into the dermis and epidermis. This leads to local transfection of the skin cells and expression of the protein encoded by the DNA. This can induce vaccine-specific immune responses through both priming and cross-priming of antigen-presenting cells [81,125,126,127]. The first study on DNA tattooing was reported in 2005 and showed strong instigation of cellular responses as well as protective humoral immunity, which have been attributed to the high number of antigen-presenting cells, as well as the adjuvanting effect of thousands of perforations [126]. Since this initial study, DNA tattooing as a means of vaccination has been investigated for a range of pathogens, including HPV, HIV, Lyme disease, melioidosis, and tuberculosis [128,129,130,131,132,133,134]. Numerous groups have considered optimising the tattooing protocol. These optimisations have included increasing the duration of puncturing and the length of microneedles, as well as the DNA concentration [125,135].

Notably, DNA tattooing has been associated with overall lower transgene expression levels but higher or equal levels of immune responses in comparison with intramuscular vaccination [129,136]. The plasmid DNA appears to suffer little damage as a result of the tattooing process, so it is unlikely that this is the root cause of the lower expression [137].

Despite much promising data, tattooing has struggled to outperform alternative techniques, when compared directly. For example, in a study with intranasal application as a comparator, C57BL/6 mice were tattooed with 20 µg of a *B. pseudomallei* flagellin DNA vector and showed an insignificant reduction in bacterial loads in lungs, whilst an intranasal application showed over a 1000-fold improvement in the bacterial load in lungs, blood, liver, and spleen [133]. Similarly, a study comparing the approach to subcutaneous immunisation in mice showed that a H56-enhanced DNA vaccine for tuberculosis applied by tattooing did not result in a reduced bacterial load in the lungs or spleen, but the BCG vaccine applied subcutaneously did provide protection [131].

Preclinical trials in rhesus macaques showed a 10- to 100-fold increase in the T-cell responses against HIV antigens from DNA-tattooed macaques compared to those injected intramuscularly, which did raise great hope for clinical trials in humans [138].

Results from the first clinical trial (NTR4607/NL4474 on trialregister.nl) with DNA tattooing in humans were published in 2017. Patients were given six vaccines by DNA tattooing for HPV16. Although no adverse events were observed, only low immune responses and no clinically useful responses could be detected [128].

To improve the delivery and immunogenicity, there have also been efforts to combine electroporation (see Section 5.3.4) with DNA tattooing. This combinatory approach used a non-viral PEGylated polymeric poly(amido amine) (PAA) gene carrier (PEG-PAA/DNA polyplex) and showed a 20-fold enhancement in antigen expression in ex vivo human skin [85].

Tattooing has also been used as a direct means of delivering peptide vaccines. In a pre-clinical model, where Wilms tumour gene 1 peptide (122–140) and CpG adjuvant was administered by tattooing in combination with i.p injected anti-TGFβ mAb 1D11, an antitumour effect could be observed, with significantly retarded tumour growth of TRAMP-C2-induced tumour cells after 32 days compared to control mice that received PBS [139].

Despite the use of existing devices, which could allow for the quick implementation of the technique, it is not clear what the future holds for this technology, as it does little to address issues of pain and patient acceptance (i.e., the cost). Hence, a clear and robust improvement in the efficacy (i.e., the benefit) would need to be demonstrated to encourage widespread adoption, and to date, this has not been shown in humans.

### 5.2. Jet and Ballistic Delivery

Completely needle-free methods have been investigated for transcutaneous delivery. These have included particle-mediated epidermal delivery and liquid jet delivery.

#### 5.2.1. Gene Gun

Particle-mediated epidermal delivery (PMED) uses ‘gene guns’ to deposit particles of coated gold beads with a diameter from 1–3 μm in the epidermal layer of the skin [83].

Experiments performed since 1993 have suggested gene guns may be a very effective method to deliver DNA into the skin, having increased reproducibility of induction of immune responses compared to intramuscular immunization in immune-competent mouse models, such as BALB/c and SJL/J [140,141,142,143]. These promising results were also bolstered by the first-in-human study, which showed a protective antibody response to hepatitis B in a study that used the PowderJect^TM^ XR1 device (PowderJect, Oxford, UK) and 1–4 µg of a plasmid optimised to express hepatitis B surface antigen. Although above protective levels, the antibody titres were still 4–10 fold below recombinant protein vaccines and have yet to translate to the clinic [144].

In a comparison of gene gun DNA vaccine administration, biojectors, and intramuscular injection for HPV DNA vaccines, gene guns also appeared to be the most potent method for DNA administration [145]. Specifically, vaccination of C57BL/6 mice with E7/HSP70 encoding DNA plasmid showed that dosing with 50 µg/mouse by needle and syringe, 50 µg/mouse by Biojector 2000 (Bioject, Tigard, OR, USA), or just 2 µg/mouse by gene gun resulted in the highest number of IFN-γ secreting E7-specific cells (375.5/(3 × 10^5^) vs. 445.5/(3 × 10^5^) vs. 832.5/(3 × 10^5^) splenocytes) being present after gene gun vaccination. After challenge with 1 × 10^4^ TC-1 E7-positive tumour cells and subsequent vaccination through the different routes, gene gun-vaccinated mice had the lowest number of pulmonary nodules, followed by the Biojector-vaccinated mice, and finally the needle and syringe-vaccinated mice [145].

Furthermore, in a non-pathogen-related cancer vaccination study using D2F2/E2 mouse tumours, the gene gun provided better tumour protection at day 25, after two immunisations with a HER2/neu-coding plasmid at day 0 and day 15. Specifically, 60% of mice immunised with a gene gun were still tumour free after 140 days, whilst only 25% of the mice immunised by a jet injector were tumour free. This was supported by IFN-γ ELISpots, which showed that gene gun-treated mice had TH_1_-polarized HER2/neu-specific CD8+ T-cell responses compared to no detectable signal for the injector-treated mice, as well as higher IgG, IgG1, IgG2a, and IgG2b signals detected by ELISA for gene gun-treated mice [146]. In other studies, electroporation and gene guns were shown to produce similar levels of antibodies in a pre-clinical mouse study of an Alzheimer’s disease vaccine [147]. However, subsequent research, which compared gene gun with electroporation and intramuscular injection of DNA vaccines in BALB/c mice, showed that gene gun predominantly induced a Th2-dominant immune response, compared to a Th1-type induction by the other methods, implying that the gene gun stimulates a humoral response and prevents initiation of a Th1 immune response [148,149].

This was followed up by research showing that electroporation of a DNA vaccine encoding HPV antigens resulted in higher cytotoxic CD8+ T cell responses and higher levels of circulating protein compared to gene guns [150]. DNA-coated microneedles were also able to elicit similar cytotoxic T lymphocyte levels when compared to the more invasive and painful gene gun [102]. A phase 1b clinical trial (NCT00349037) showed that the gene gun approach could elicit protective immunity against influenza [151]. However, the efficacy of the DNA vaccination was lower than those reported for trivalent-inactivated influenza vaccines delivered by N&S (41–53% vs. 69–70%) [151,152]. Since this clinical trial, studies have been published that investigated methods to improve the immunogenicity of the gene gun approach. Pre-clinical work in macaques showed that gene gun co-delivery of the GM-CSF gene could help induce antibody and T cell responses in the lungs and gut against influenza [153]. Co-administration of the chemokine CCL19 was also shown to improve tumour protection [154]. Systemic administration with synthetic oligodeoxynucleotide carrying immunostimulatory CpG motifs in combination with gene gun vaccine administration was also demonstrated to improve antitumour benefit [155].

A remarkable study was done by Aps et al., where it was shown that killed bacterial spores from *B. subtilis* treated with 2mM dioctadecyldimethylammonium bromide (DODAB) could be used as low-cost microparticles for DNA delivery by the gene gun, removing the need for gold. C57BL/6 mice were treated twice with either gold particles or spores coated with luciferase plasmid DNA. Results showed that 2 µg of DNA on killed spores resulted in half the luminescence compared to gold particles. However, by doubling the luciferase plasmid DNA to 4 µg on the killed spores, similar levels of luciferase expression could be achieved. This translated to immune responses where the same level of antibody titres, IgG1/IgG2a ratio, and IFN-γ-producing CD8+ T cells were observed after two vaccinations with 2 µg of pgDE7 h DNA-coated gold particles and 4 µg of pgDE7 h DNA-coated spores [77].

A combinatory approach of intramuscularly delivered protein and gene gun-delivered DNA for dengue vaccination has shown protective immunity in macaques [76]. In another macaque study, where the DNA sequence of heat-labile enterotoxin from *E. coli* was added to the influenza DNA vaccine, high titred antibodies, cross-reactive T cell responses, mucosal responses, and rapid viral clearance after challenge were observed. However, no broadly neutralising antibody level was induced in a neutralisation assay [156]. Regulatory T-cells have been implicated in diminishing antigen-specific effector T-cell responses after DNA vaccination by the gene gun and may explain why gene gun approaches have struggled to transition to the clinic [157]. In addition, the patient experience is not one that is free of pain; in one study with ND10 gene guns by PowderMed Vaccines, 48–56% of volunteers reported pain at the injection site [158]. Despite the high hopes raised by preclinical studies, gene guns will need to improve their immunogenicity in humans to be able to progress further in clinical trials and achieve marketing authorisation.

#### 5.2.2. Jet Injection

Jet injection is a needle-free method that uses a stream of high-pressurised liquid to penetrate the stratum corneum. Multiuse-nozzle jet injectors have been in use since the 1960s for the delivery of the intradermal vaccination for smallpox and the bacille Calmette-Gueri (BCG) vaccine for tuberculosis amongst others [159]. In order to avoid cross-contamination, these jet injectors were superseded by disposable-syringe jet injectors. Some of these relied on a manual firing mechanism, e.g., for use in developing countries, and others relied on a disposable gas canister to create the jet [83].

Many of these more novel jet injectors are being assessed in clinical trials and have shown comparable immunogenicity to needle and syringe techniques. Devices assessed in clinical trials include the Injex30 (Injex Equidyne, Oxford, UK), Biojector 2000 (NCT00109629) (Bioject, USA), Bioject ZetaJet (NCT01407497) (Bioject, USA), the PharmaJet Stratis (NCT01688921) (PharmaJet, Golden, CO, USA), and the TriGrid electroporation systems (NCT02589795) (Ichor Medical Systems, San Diego, CA, USA) [160,161,162,163,164,165].

The Biojector 2000 is an FDA-approved device with a long clinical history and was originally used for the administration of medication, such as midazolam, lidocaine, heparin, and morphine, but has since also been used for vaccine and DNA injection [161,166,167,168,169]. The Biojector 2000 can be used for injection in the intramuscular, subcutaneous, or intradermal compartment [161]. The subcutaneous administration with the Biojector has been associated with a decrease in injection site reactions and has been rated as significantly easier to use compared to needles by self-administrating patients [170]. In a clinical trial to assess the efficacy of a fractional dose of the inactivated polio vaccine delivered intradermally with the Biojector 2000, over 90% of the infants seroconverted after two fractional doses [171]. Another study in Oman corroborated these findings and showed that with a fractional dose (1/5th) of inactivated polio vaccine applied with the Biojector 2000, over 90% of infants seroconverted; however, they did have significantly lower antibody titres compared to the full dose injected intramuscularly [172]. However, a similar study performed in Cuba with a large cohort of 364 infants showed that the fractional dose of inactivated polio resulted in a significantly reduced seroconversion compared to the full dose applied with the Biojector [173].

The Bioject ZetaJet is also FDA approved and can be used for DNA vaccine delivery intradermally and has been used in both preclinical and clinical trials for a variety of vaccines [174]. The ZetaJet elicited neutralising antibodies against Rift Valley fever virus (RVF) in a rat model following delivery of 1 × 10^3^–1 × 10^5^ PFU of live recombinant RVF MP-12ΔNSm vaccine [175]. However, in a HIV vaccine clinical trial (NCT01407497), three HIV-DNA immunisations only resulted in IFN-γ response rates above 55 SFCs/million PBMCs in 9/17 participants for Gag and 0/17 participants for Env. A boost of HIV-MVA was needed to elicit IFN-γ response rates above 55 SFCs/million PBMCs to Gag for 93% of participants and Env for 87% of participants [164].

A range of other devices have been developed and trialled in recent years. For instance, Genetronics Inc (San Diego, CA, USA) patented an injector pen, which uses a spring mechanism to apply needle-free intradermal injection of a liquid [176]. The Injex30 provided comparable antibody titres to subcutaneous N&S injection in a study with mice vaccinated against hepatitis B surface antigen [163]. A pyro-drive jet injector (Actranza™, DAICEL Corporation, Osaka, Japan) has been shown to allow for the adjustment of injection depth and has demonstrated potential to induce antibodies in rats [177].

The Pharmajet Stratis, a disposable-syringe jet injector, has been assessed in a clinical trial (NCT02253407) in India. It was shown that similar levels of seroconversion were observed after measles, mumps, and rubella (MMR) vaccination by jet injector as with needle and syringe injection in 170 toddlers 35 days post-injection, as well as similar adverse effect rates [178]. However, another trial (NCT02409095) in India with the same disposable-syringe jet injector but for DTP-HB-Hib vaccination had to be prematurely terminated after a higher frequency of injection site reaction was observed in those treated with the jet injector compared to those injected by needle and syringe. In particular, discoloration, injection site bleeding, and nodules were observed and the majority of moderate and severe local reactions occurred in the jet-injected group compared to the needle and syringe group, but all adverse reactions resolved without any sequelae [179]. These results showcase that, although disposable-syringe jet injectors may be effective, they need to be carefully designed in conjunction with the vaccine to avoid adverse effects.

As the interest in DNA vaccines increases, methods to combine electroporation with intradermal jet vaccination are gaining traction. The needle-free jet injector, the Pharmajet Tropis (PharmaJet, USA), has been tested for delivery of dengue DNA vaccines and compared to the TriGrid electroporation system from Ichor medical systems. Both systems use needle-free jet injection, but the TriGrid has combined this with electrodes to increase cell membrane permeability and thereby aid DNA delivery to the nucleus and improve the resulting immune responses [35,180]. Notably, TriGrid has also recently announced a collaboration with the Naval Medical Research Centre to deliver a DNA vaccine against the spike protein of SARS-CoV-2 and testing has progressed to non-human primate models [181]. Another example is Derma Vax™, a DNA vaccine electroporation system developed by Cyto Pulse Sciences Inc., Glen Burnie, MD, USA. This has progressed through phase II clinical trials for HIV vaccination (NCT01697007) and has shown increased response rates to DNA-encoded Gag when the vaccine was delivered using a Bioject^®^ Zetajet, a liquid jet injection device, and Derma Vax™ was used to electroporate [182]. Enthusiasm for these encouraging results is perhaps tempered by the complexity and cost involved in combining two technologies.

A study combining the jet injector with magnetic DNA transfection has shown some benefit in transfecting rats with DNA encoding luciferase, but this work has not yet progressed to the clinic [183].

Despite the long history of jet injector use, recent adoption rate has been low. A peak in their use was seen in the US military between 1947 and 1965 but was rapidly decreased after evidence of cross-contamination was uncovered. The introduction of disposable cartridges has resulted in a renewed interest in jet injectors and multiple devices are marketed now, but these are still not widely utilised. Possible reasons for this have included the pain experienced by patients, bleeding, oedema, the costs associated, and the ease of use of the devices. Further verification is also needed regarding the penetration and dispersion mechanism and the effect of the mechanical tissue properties for jet injectors to become more widely adopted as a vaccination technique [184,185].

### 5.3. Permeabilization of the Skin

Direct alteration of the skin to make it more permeable to vaccines, thereby circumventing the need for needles, has been attempted in numerous ways, including thermal ablation, chemical enhancer addition, abrasion, electroporation, ultrasound, and iontophoresis.

#### 5.3.1. Thermal Ablation

Thermal ablation devices use lasers to create aqueous micropores in the skin, after which a drug or vaccine can be applied. The lack of contact of the perforating device with the skin reduces cross-contamination risks and the utilisation of laser scanning technology allows for flexibility in the depth, number, and density of the created micropores. One of the early laser devices was named P.L.E.A.S.E.^®^ (Precise Laser Epidermal System) and was developed by Pantec Biosolutions, Ruggell, Liechtenstein. Weiss et al. conducted a study in BALB/c and C57BL/6 mice that targeted a variety of layers of the skin using the device and compared immune responses to subcutaneous injection. This showed T-cell polarisation could be biased: subcutaneous injection resulted in FITC-dextran (2000 KDa) being predominantly taken up by CD207-CD11b- dendritic cells, whilst laser-mediated poration resulted in antigen uptake by CD207-CD11b+ dendritic cells. This implies that laserporation favours antigen uptake by inflammatory dendritic cells, which specializes in activation of CD4 T-cell and humoral immunity. Notably, no thermal damage was observed, and the skin epithelium had completely recovered within 2 days [186]. This agreed with previous human trial results on ablative fractional laser pretreatment, showing that thermal ablation was well tolerated, with 12 patients rating thermal ablation 3 on average on a pain scale of 0 to 10. However, pain scores ranged from 1–9 for individual participants [187]. The technology has continued to progress and a clinical trial (NCT02988739) sponsored by Pantec Biosolutions AG, investigating the utility of the P.L.E.A.S.E. for the seasonal influenza vaccine Intanza, has been completed, although the results of this study are yet to be published [188].

An alternative is the UltraPulse^®^ Fractional CO_2_ Laser (Lumenis Inc., Borehamwood, UK) that limits potential damage induced by an ablative fractional laser and creates conical-shaped microchannels. It has performed well when compared to tape stripping and elicited a humoral immune response to OVA, which was significantly higher than that achieved with tape stripping in a BALB/c mouse model (titres up to 1068 for AFL vs. 14 for tape stripping) [189]. These approaches are still reliant on a two-step process, whereby pores are created and then vaccine is applied and must diffuse through the channels created.

In the past few years, the attention of researchers using lasers has shifted to utilising these lasers as adjuvants for vaccination [190]. Four types of laser have been studied: Ultra-short-pulsed lasers (UPLs), continuous wave lasers (CWLs), non-ablative fractional lasers (NAFLs), and ablative fractional lasers (AFLs), which cause varying degrees of damage [191]. NAFLs and AFLs are suspected of instigating the release of damage-associated molecular patterns. This stimulates the migration of antigen-presenting cells to the site [188,192]. In CWLs, reactive oxygen species (ROS) are thought to play a crucial role. This was shown by firstly demonstrating the generation of ROS in both cultured BMMCs and keratinocytes. Subsequent experiments showed that the addition of an ROS scavenger (NAC) decreased the effect the CWL had on the migration of dendritic cells, as quantified by an FITC paint technique. In a Ccr7^−/−^ mouse model, lacking the ability to traffic migratory dendritic cell populations from the peripheral tissue, no enhancement of antibody titres for the CWL could be observed, whilst an increase in antibody titre was seen in C57BL/6J mice upon CWL [193].

Although in its early stages, thermal ablation does offer significant advantages in terms of safety and ease of use.

#### 5.3.2. Chemical Enhancers

Chemical enhancers modify the stratum corneum to make it more amenable to the delivery of agents into the dermal layers. Ideally, they should be non-toxic, non-allergenic, have a rapid and predictable duration of activity, function unidirectionally, and be compatible with the structure and mode of action of drugs and vaccines [194]. It is clear that there may be difficulties in meeting these requirements whilst also identifying a chemical that has often contradictory properties, which allow it to compromise the integrity of the stratum corneum. Chemical enhancers have been predominantly used for drug delivery in the literature, but they have the potential to also be applied to the delivery of vaccines.

One of the early mechanisms tested for improving permeation was hydration of the stratum corneum. Soaking the skin in water or preventing transepidermal water loss can result in the water content increasing to up to 400% of the tissue dry weight. This results in the stratum corneum having a water content in equilibrium with the epidermal cells below [195]. Utilising this mechanism, hydrogel patches can improve the immune response outcomes. By hydrating the skin, tetanus and diphtheria toxoids could elicit antibodies on the same level as subcutaneous vaccination, predominantly Th2-type immune responses and effective immune memory. Vaccination of hairless mice with a hydrogel patch for 24 h resulted in complete survival after tetanus toxin challenge [196]. In a follow-up study, volunteers were treated with a hydrogel patch containing antigen for 24 h and then boosted with the same system after 140 days. The antibody titres increased significantly, although only in a subset of the participants (for tetanus toxoid: 7/27 and for diphtheria toxoid: 9/27). As most volunteers had been vaccinated for tetanus and diphtheria as infants, most subjects already had protective levels at the start of the clinical trial. The few participants that did not already have protective antibody titres showed no increase in their level and it is suggested that the hydrogel patch may induce little immunity as a primary vaccination strategy in humans [197].

Dimethylsulphoxide (DMSO) is one of the most well-known and studied penetration enhancers, as it allows for the penetration of both hydrophilic and lipophilic compounds [195]. DMSO is an organic solvent and interacts with the lipids in the stratum corneum, leading to the partial extraction of these lipids and an increase in lipid fluidity [198,199]. A 10% DMSO solution has been shown in a K14-VEGF mouse model to enable the increased penetration of DNA into the dermis. One day after the final treatment of three daily topical applications of pLacZ in DMSO, mice showed a near four-fold increase in transgene expression compared to the low level achieved by application of pLacZ in MilliQ water alone. This also proved an effective method to deliver an interleukin-4 plasmid for the treatment for psoriasis [200]. It is possible this method could be applied to DNA vaccines as well. Disadvantages of DMSO are that a high concentration can cause erythema, scaling, stinging, and burning sensation. This has led to some chemically related, but potentially less harmful, compounds being investigated, including dimethylacetamide (DMAC) and dimethylformamide (DMF) and decylmethylsulphoxide (DCMS). DMF was shown to result in irreversible damage to the stratum corneum, but DCMS was reported to have only a transient effect on the skin and can be used in hydrophilic permeants [195,201].

Azone was the first compound designed as a skin penetration enhancer, and was still under recent investigation for the transdermal delivery of thymoquinone, a compound with potential therapeutic effects [202,203]. Similar to pyrrolidones and fatty acids, its mechanism is probably the disruption of the lipid domains in the stratum corneum [195]. However, pyrrolidones can form reservoirs in the skin, allowing for a sustained effect. Reports showed a peak urine concentration for a pyrrolidone metabolite product 10 h after dermal exposure to an aqueous dilution of 5-hydroxy-N-methyl-2-pyrrolidone [204,205]. However, pyrrolidones have failed to achieve clinical translation due to reported adverse effects [195,206].

A variety of essential oils have also shown a permeation-enhancing effect. A recent example is white mustard (*Sinapis alba* L.) seed volatile oil (SVO), which was more effective ex vivo than azone in enhancing the penetration of three model drugs, 5-Fluorouracil, Osthole, and Paeonol, into rat skin, whilst causing less skin damage [207].

Other classes of enhancers include surfactants and phospholipids. Surfactants work by solubilising the lipids in the stratum corneum and have been used to improve the delivery of membrane antigens from *Salmonella enterica* into the skin in vivo [208]. Phospholipids can be used as carriers of antigens. Flexible liposomes have generated antibody responses against OVA by bypassing the skin barrier via delivery by the hair follicles in mice [209]. Cationic lipid nanoparticles can also specifically enhance the DNA delivery. The mechanism of these nanoparticles relies on charge interactions between the nanoparticles and the skin, and enhanced delivery of an enhanced green fluorescent protein (EGFP) plasmid in vivo in BALB/c mice was shown to be on par with a lipid vesicle delivery system [210].

Finally, thermoresponsive nanogels, which can shift from hydrophilic to hydrophobic, provide a triggered increase in penetration through the stratum corneum and may therefore provide an inducible penetration enhancer [211].

To date, however, no penetration enhancers have entered clinical trials for vaccine delivery and further research both on the mechanism, prevention of adverse effects, and effect on vaccine formulation, some of which may have a lipid bilayer coat and will therefore be sensitive to their mechanism of action, will be needed.

#### 5.3.3. Abrasion

Abrasion of the stratum corneum is another method of circumventing the impermeable outer layer of the skin. This can be achieved via the use of tape stripping, sanding, a razor, a toothbrush, or microdermabrasion. Application of a drug or patch to the abraded area can then allow easier passage of the drug into the dermal layers.

Although all these approaches create concerns around patient pain and compliance and possible infection, some interesting effects have been shown. For instance, CpG oligodeoxynucleotides applied topically after tape stripping improved T-cell persistence in the local epidermis and increased circulating memory cells, showcasing the potential for direct delivery of nucleotides by abrasion [212]. However, in a human study, the topical application of adenovirus-vectored influenza vaccine in combination with shaving and toothbrush strokes could not match the immune responses generated by nasal vaccine [213].

Microdermabrasion is an FDA-approved cosmetic procedure that damages the stratum corneum with abrasive particles under vacuum. This damage causes an inflammatory response, which may be beneficial for vaccines [214]. In a preclinical study in macaques, microdermabrasion enabled the generation of antibodies against a vaccinia virus that had been topically applied post abrasion [215]. A modified vaccinia virus expressing the full-length spike protein of SARS-CoV-2 is also under investigation as a COVID-19 vaccine and has elicited neutralising antibodies in Balb/C mice [216].

A more recent abrasion technique is an abrasive gel made from star-shaped particles (STAR) particles, which are 17-mm-sized particles with micron-sized protrusions that have been found to be well tolerated by patients. These particles form microscopic pores through which drugs or vaccines can be delivered into the skin. A study in mice treated with fluorouracil in combination with STAR particles showed they had significantly smaller tumour volume compared to mice treated with fluorouracil alone after 12 days, as well as a longer median survival time. They also showed that topically applied tetanus toxoid vaccine with STAR particles was able to elicit IgG, IgG1, and IgG2a antibodies of equal magnitude to intramuscularly delivered tetanus toxoid vaccine, although a 40 to 200 times higher concentration flocculation units of toxoid per mL was used for the topically applied vaccine. Upon challenge after one year, all STAR-vaccinated mice survived three challenges with tetanus toxoid, whilst only one out of three intramuscularly vaccinated mice survived after the second challenge, and none survived a third challenge [217].

These newer approaches may enable abrasive techniques to enter the clinic, especially as it utilises previously approved devices or cheaply produced consumables that are widely applicable. However, concerns remain around patient pain and compliance and possible infection after abrasion techniques and may require strict hygiene protocols to avoid unintended exposure to pathogens. Notably, the technique when used in isolation still relies on passive concentration-driven movement of agent into the target skin area.

#### 5.3.4. Transfollicular Vaccination

Hair follicles provide a natural route to bypass the stratum corneum and are particularly attractive as a vaccination site due to the accumulation of Langerhans cells and dendritic cells around hair follicles, as well as their reservoir properties [218]. As early as the 1960s, follicular ducts were proposed as the dominant diffusion route during the initial transient diffusion [219]. However, significant further investigation into this route only took off in the 1990s [220]. In 1999, Fan et al. showed that normal hair follicles are required for the transgene expression in mice from a hepatitis B antigen-encoding plasmid topically applied to the skin. It was also demonstrated that only in C57/BL6 mice, where the plasmid was applied to normal skin instead of on nude mice skin grafts, an antibody response could be detected [221]. To optimally utilise the transfollicular route, skin surface stripping or tape stripping is often combined to enable the opening of the hair follicular duct before vaccine application. Mahe et al. showed that 40- and 200-nm fluorescent particles applied onto tape-stripped skin of C57BL/6 mice penetrated particularly through the hair follicle openings. They also showed that topically applied 100 µg of OVA protein with cholera toxin produced similar antibody responses as 100 µg of plasmid encoding OVA [222].

Cyanoacrylate (glue) skin surface stripping, which removes approximately 30% of the stratum corneum, allows for follicular penetration of vaccines by removing lipids, cell debris, and keratinised material that may have accumulated in the follicular openings [223]. This method has been applied in clinical trials for vaccination with inactivated influenza virus vaccine (NCT00261001) and GTU^®^MultiHIV B clade DNA vaccine (NCT02075983) [224,225,226]. In the influenza vaccine trial, a noticeable absence of neutralising antibodies was observed compared to intramuscular injection but also an increase in CD8+ effector T cells. This provided further proof of the role different delivery methods play in shaping the immune response. In the GTU^®^MultiHIV B clade DNA vaccine trial, no humoral response was observed for intramuscular, intradermal, or transcutaneous vaccination, but a shift to a more Th-17 cell response was observed for intramuscular vaccination in combination with transcutaneous vaccination applied with cyanoacrylate skin surface stripping [225]. However, the use of tape stripping does require a good hygiene protocol to prevent unwanted contamination.

Nanoparticle-based vaccines that utilise this route have been suggested as a method to improve the targeting of antigen-presenting cells in the skin. Surfactant-based inverse micellar sugar glass nanoparticles (IMSG NPs) encapsulating OVA showed increased follicular uptake compared to non-encapsulated OVA in C57BL/6 mice [227]. Poly(lactide-co-glycolide) (PLGA) and chitosan-coated PLGA (Chit-PLGA) nanoparticles similarly showed a 2.85 ± 0.6 and 2.33 ± 0.52 fold improvement in the uptake of FITC-OVA compared to a FITC-OVA solution in pig ears [228]. However, it does appear that the addition of adjuvants is required to elicit a strong humoral and cellular immune response. Mittal et al. showed that the addition of the adjuvant bis-(3′,5′)-cyclic dimeric adenosine monophosphate was necessary to get significantly higher antibody titres compared to a dose-matched OVA solution in C57BL/6 mice [229].

Overall, there is clear potential for transfollicular immunization. However, if the use of tape stripping is pursued, it may make it difficult for the technology to enter the clinic, as it does result in pain for patients, requires strict hygiene protocols to prevent the entry of unwanted pathogens, as well as cost significant amounts of time of health care workers. Further improvements in the formulation that circumvent the need for tape stripping, such as nanoparticles, as well as improve immunogenicity, would help this technique transition into the clinic.

#### 5.3.5. Electroporation

Electroporation is typically used to increase the immunogenicity of DNA vaccines, particularly by improving the delivery of DNA to the cell nucleus. The stage of transfer into the nucleus has been shown to be particularly inefficient for plasmid DNA [230]. In laboratory settings, electroporation has long been used to transfect bacteria and mammalian cell lines. Electroporation relies on the generation of transient pores, induced by the application of an electric field. These pores allow the DNA to enter cells in the target tissue [231]. Electroporation has been applied to enhance intramuscular DNA uptake, as the muscle seems a particularly attractive target for electroporation, perhaps because of the unique electrophysiology of muscle cells [232]. However, electroporation has also been used for intradermal DNA uptake in humans, mice, pigs, and non-human primates [233,234,235,236]. Clinical trials investigating the utility of DNA vaccines delivered by electroporation into the skin have been conducted for a variety of illnesses, including prostate cancer, influenza, and HIV, and overall have shown favourable safety profiles, which have encouraged further development [237].

The potential of dermal electroporation to enhance the efficacy of DNA vaccines has been probed in multiple trials, with varying levels of success. For example, a phase I clinical trial of a DNA vaccine for dengue fever applied with the Biojector^®^ 2000 (NCT00290147) failed due to a lack of immunogenicity [238]. Following this, studies were repeated in macaques with alternative methods of vaccine delivery. Subsequent research in macaques has shown that the addition of electroporation to a DNA vaccine encoding pre-membrane (prM) and envelope (E) genes of dengue 1, 2, 3, or 4 viruses can improve both the IFN-γ and the antibody responses generated. A year post vaccination, the macaques vaccinated by electroporation had significantly fewer days of RNAemia compared to non-vaccinated controls upon challenge [35]. Dermal electroporation was also the method used in a successful study of a DNA vaccine against Lassa virus tested in macaques, where two vaccination doses resulted in complete protection against intramuscular injection of 1000 pfu of Lassa virus [239]. Later work showed that a two-dose vaccine regimen provided specific cellular responses and antibody responses with a mean titre of 727 [240]. Notably, the COVID-19 DNA vaccine being developed by Inovio is being delivered intramuscularly using the electroporation device CELLECTRA^®^ (Inovio, San Diego, CA, USA) [241]. This vaccine is based on a previous phase I clinical trial on Middle East respiratory syndrome (MERS) (NCT02670187) also utilising a Cellectra device, which elicited immune responses in 85% of volunteers [242]. The COVID-19 vaccine has shown T-cell responses and neutralising antibody responses in rhesus macaques [243] and has progressed to phase I/II clinical trials (NCT04447781/NCT04336410), where ID injections are combined with Cellectra^®^ 2000 [244].

However, it should be mentioned that currently a major adverse effect of electroporation is the pain experienced, which has been deemed worse than receiving a needle and syringe injection by patients. In a study (NCT00721461) with the Medpulser^TM^ DDS (Inovio, USA) to establish tolerability, 24 patients were asked to rate the pain intensity from 1 (mild) to 5 (excruciating) and nearly 40% rated it at 3 or above and 50% had electroporation-site pain recorded as an adverse effect [245].

In another clinical trial (NCT00563173) investigating hepatitis C vaccination through electroporation with the same Medpulser^TM^ device (Inovio, USA), 12 participants with chronic hepatitis C virus infection were asked to rate the pain from injection and electroporation for four treatments each on a 0–10 scale, with zero representing no pain and 10 representing excruciating pain. Injections were rated between 0–5, whilst electroporation was rated between 2 and 8 [246].

The EasyVax^TM^ device (Cellectis Therapeutics, Paris, France), a dermal electroporation device, was also tested for tolerability. Of the 10 subjects, none reported pain above 3 on a similar 0–10 scale. However, some subjects still described it as painful and would ‘prefer needles’, although others saw it as less painful and described less residual pain compared to traditional vaccination [247]. This challenge will need to be addressed to ensure patient compliance and acceptance of this novel technology.

To date, there are no DNA vaccines approved for human use, but there are four veterinary vaccines that are commercially licenced: West Nile–Innovator^®^ (equine), Apex-IHN^®^ (salmon), LifeTide^®^ SW5 (swine), and Oncept™ (canine) [248]. Most of these vaccines are injectable, but LifeTide^®^ requires the addition of electroporation [249]. Oncept™ is delivered via jet injection with the Vet Jet™ (Merial Ltd., Duluth, GA, USA) [250].

The push for novel vaccines to tackle the COVID-19 crisis may help in stimulating the transition of the technology from something viewed as being only applicable to animals to a viable option for humans. Notably, the rapid progress of Inovio’s COVID-19 DNA vaccine, which is in phase I/II trials (NCT04447781/NCT04336410), as well as the progress made by Cadila Healthcare Ltd., who have just started a phase I/II clinical trial (CTRI/2020/07/026352) where a DNA vaccine is injected intradermally, may stimulate developments in intradermal DNA vaccines. More broadly, acceptance of nucleotide-based vaccines will likely benefit from the many vaccine candidates in development. On the 13th of August 2020, 10 out of 29 candidate vaccines in clinical trials for COVID-19 utilised a DNA or RNA vaccine platform. Although, it should be noted that the majority of nucleotide-based vaccines in clinical trials currently depend on lipid nanoparticles platforms that are intramuscularly injected, most notably Moderna’s lipid nanoparticles (LNP)-encapsulated mRNA [244,251]. The pandemic is also driving forward more novel nucleotide-based vaccine technologies, such as the self-replicating RNA vaccine formulated with a lipid inorganic nanoparticle (LION) [252].

Electroporation has also been used in combination with many other techniques, including iontophoresis, jet injection, DNA tattooing, and microneedles [85,112,182,253]. Such combinations would have to demonstrate step-change enhancements to warrant the extra manufacturing, administration and regulatory complexity involved in bringing the approach into clinical practice.

#### 5.3.6. Iontophoresis

Iontophoresis uses an electrical field to deliver drugs and vaccines into the skin and is particularly effective for charged and polar molecules [254]. Two electrode patches are applied to create an electric circuit through the skin and by applying a small electric current across the skin, drug permeation is increased. The electrode with the same charge as the drug is used to drive the charged drug through the stratum corneum into the skin [255]. Electrorepulsion and electroosmosis have both been named as the underlying mechanisms [256].

In common with the use of cavitation (see below), delivery is no longer fundamentally linked to passive diffusion and concentration gradients, as a force for the active propulsion of drugs is applied. However, the approach is by no means drug agnostic and relies on the agent being delivered having particular charge characteristics. In the context of vaccines, Xu et al. showed that iontophoresis induced a significant improvement in the retention and total amount of hepatitis B vaccine in the skin of a rat in a Franz diffusion cell, compared to diffusion alone. Up to a 6.6-fold improvement of the permeation of the hepatitis B vaccine was observed with iontophoresis for an initial concentration of 46 μg/mL in the donor compartment [257]. In 2020, a study in HWY hairless rats, where psoriasis was induced with imiquimod, showed that etanercept delivered by iontophoresis reduced the transcript levels of IL-6 24 h after two treatments. The etanercept on a nonwoven fabric with a surface area of 2.25 cm^2^ was attached to electrodes and the circuit was completed with a nonwoven fabric with PBS. The epidermis thickness also decreased most after iontophoresis treatment for psoriasis, whilst the thickness of the epidermis did not change significantly after subcutaneous injection of etanercept [258].

Work has also been performed to investigate lipid nanoformulations for effective delivery by iontophoresis. For instance, OVA-loaded liposomes and silver nanoparticles have been effectively delivered into the epidermis by iontophoresis and showed a 92-fold improvement, compared to passive delivery, in vitro into the viable epidermis (passive penetration: 0.05 ± 0.02 μg/cm^2^, iontophoresis: 4.61 ± 0.12 μg/cm^2^). These OVA-loaded liposomes and silver nanoparticles could then induce anti-OVA antibodies and encourage the differentiation of immune cells in a BALB/c mouse model [259]. Another ex vivo study into doxorubicin-loaded solid lipid nanoparticles showed that after 6 h, significantly more doxorubicin-loaded solid lipid nanoparticles were present in the viable epidermis of pig ears after iontophoresis compared to passive diffusion (3250.4 ± 190.2 ng/cm^2^ vs. 515.1 ± 49.8 ng/cm^2^) [260].

The approach has also been pursued for use in cancer vaccination. In one study, the cancer antigen gp-100 peptide KVPRNQDWL was loaded into a nanogel, which was placed on the skin, and a moistened fabric was placed 1 cm away and an anode and cathode were connected to each, respectively. This resulted in accumulation of the antigen in the epidermis and migration of Langerhans cells into the iontophoresis-treated skin. The vaccination strategy managed to suppress tumour growth of previously implanted B16-F1 tumour cells compared to a non-treated control and iontophoresis with peptide or saline only. This shows that effective vaccination in vivo can be achieved in this Hos:HR-1 mouse model [261].

Ionotophoresis has also been tested for the delivery of DNA vaccines for cancer treatment. CpG oligodeoxynucleotides with antitumour activity have been delivered into the skin with iontophoresis and were shown to induce immune responses, including proinflammatory and Th1-type cytokines, and antitumour activity in mice. Specifically, B16F1 murine melanoma cells implanted into Hos:HR-1 hairless mice were shown to have retarded growth compared to non-treated animals and comparable growth to subcutaneous injections of the DNA [262]. In another study, antisense oligonucleotides for Bcl-2 delivered by iontophoresis were used to treat CD1 mice with induced skin tumours. Complexing the oligonucleotides with a PAMAM dendrimer resulted in a positively charged complex that resulted in a significantly reduced tumour volume (by 45%) when iontophoresis was used for delivery, in line with a similar reduction in Bcl-2 levels. These results were not seen following intradermal injection of the DNA [263].

In the context of drug delivery, benefit has been found by combining iontophoresis with hydrogel microneedles. When an insulin-loaded patch, hydrogel microneedles, and electrodes were applied to diabetic rats, their blood glucose levels could be decreased by 70.42 ± 1.86 % compared to just iontophoresis with a patch (39.06 ± 2.10 %) or only a patch with hydrogel microneedles (63.16 ± 2.82%). The combination of microneedles and iontophoresis also markedly decreased the time needed to reach this maximal drop from 12 h for microneedles alone to 6 h with iontophoresis and 4 h for the combination of microneedles and iontophoresis [253]. This method of combining iontophoresis with hydrogel microneedles could potentially also be applied to the delivery of vaccines. Although, the manufacturing and regulatory complexity added for the benefit gained needs to be considered.

Iontophoresis was brought to the market in the form of the Zecuity^®^ device used for the delivery of the painkiller sumatriptan. However, the product was later withdrawn due to multiple reports of adverse effects, such as scarring and burning [264]. Currently, ActivaPatch^®^ (North Coast Medical, Inc., Morgan Hill, CA, USA) and Iontopatch^®^ (IontoPatch, Paul, MN, USA) are commercially available as single-use iontophoresis patches for a variety of drugs [265]. This has been followed up by research into reusable iontophoretic patches with increased control over delivery, which has shown up to a 10-fold increase in lidocaine hydrochloride permeation in a Franz diffusion cell compared to passive diffusion [265,266]. Although currently predominantly used for drug delivery, research into DNA delivery may make it a suitable delivery technique for DNA vaccines.

#### 5.3.7. Ultrasound

Sonoporation refers to the generation of pores in cell membranes as a result of exposure to ultrasound [267]. Such pores are thought to be generated by cavitation events, which involves the expansion and collapse of gas bubbles in response to the alternating rarefactional and compressional cycles of an ultrasound wave [268]. If a bubble expands uncontrollably, it can reduce the pressure in the cavity to the point where the gas–liquid interface cannot support the inertia of the liquid around the bubble, causing it to collapse. Such events are associated with microstreaming and have been shown to increase the penetration and extravasation of free drugs in medical applications [269]. The pores created by cavitation can provide a route for the passage of therapeutic agents into cells. The approach is particularly attractive for the delivery of nucleotide-based therapies, such as RNA and DNA, which are typically very effectively excluded from cells [270]. The approach also holds potential for permeabilising the stratum corneum and allowing transport into the layers below [271].

Early studies in this field applied the approach in two steps: Firstly, the skin would be exposed to ultrasound with or without gas-bubble cavitation nuclei to create pores, and then a second stage of drug application would be performed [272,273]. Whilst encouraging results were produced, the process was somewhat suboptimal and is hard to standardise for clinical practice. Crucially, the convective flow and propulsion of drug that can be instigated by cavitation events was not utilised by temporal separation of the cavitation event and the drug exposure step, which was ultimately still reliant on passive diffusion.

More recently, ultrasound-mediated cavitation has been shown to enhance drug penetration and extravasation in cancer therapy by providing a convective stimulus to push the drug into and throughout solid tumours [274,275]. Notably, this happens without damage to the structure or activity of the drug [276,277].

A crucial step in being able to apply the approach to transcutaneous delivery was the production of a gas nuclei that would allow cavitation to be generated continuously over several minutes and thereby allow a one-step simultaneous poration-plus-delivery approach [269]. These gas nuclei were denoted ‘nanocups’ and it is theorized that the sustained microstreaming and microjetting they create in response to ultrasound can permeabilize the stratum corneum, allowing penetration into the epidermis whilst also propelling the drug/vaccine and the nanocups themselves. Using these cavitation nuclei, enhanced transdermal delivery of the model protein OVA across the stratum corneum was shown and an immune response instigated [278,279].

A further intriguing aspect of ultrasound application is the accumulating evidence that low-frequency ultrasound can function as a potent adjuvant, as it has been shown to directly activate immune cells [280]. Despite the advantages of utilizing ultrasound for transdermal delivery of vaccines, to date, no clinical trials have been conducted to establish the safety and efficacy in humans. This may be the consequence of the fact that until recently, there was a dearth of technology that would allow the generation of sustained cavitation events at relatively minor pressure amplitudes.

Notably, it has also been shown that sonophoresis has synergistic effects with the other transdermal techniques, such as microneedles and electroporation [281]. Again, the costs and complexities of bringing multimodal technologies into clinical practice may limit the translation of such combinations.

## 6. Conclusions

Research into a wide variety of techniques, being performed to tap into the full potential of transcutaneous vaccination, has been detailed here (Table 1, Figure 3). These strategies vary from microneedles, needle-free injection, and direct alteration of the skin’s properties and have shown potential in both preclinical and clinical studies. A common thread throughout the field has been the necessity to develop delivery techniques in conjunction with formulations to ensure efficacy and a need to understand the fundamental mechanisms underpinning the diversity of observed immune responses. An improved understanding of the immune responses generated will also help the field move towards developing the platform vaccination techniques that are becoming ever more relevant as we try to tackle novel and difficult diseases. Although transcutaneous vaccines are yet to become mainstream techniques, marked steps have been taken to get technologies into the clinic and gain a better understanding of the patient’s perspective. Further work will be required on the commercialisation of these techniques, the development of new medical protocols that ensure proper hygiene, and how to scale the novel devices and formulations developed in the lab. In response to the urgent need to improve patient experience, utilise novel vaccine formulation, and protect patients and healthcare workers from needle stick injuries, researchers continue to improve the technologies available and showcase a remarkable inventiveness in developing new innovative technologies.

## 7. Methods

In writing this review paper on the historical context and future potential of vaccination technologies, an initial broad search on google scholar was done for ‘transdermal’, ‘intradermal’, and ‘transcutaneous’, ‘vaccine’ delivery’, and ‘vaccination’. Based on the results, a framework for the manuscript was created. Key publications, selected based on citations and impact in the field, as well as clinical trials and examples of commercialisation of each technology, were included where possible. For clinical trials, trials with healthy volunteers were selected, unless explicitly mentioned.

## Figures and Tables

**Table 1:** An overview of different vaccination techniques for the dermal compartment, the device developed, and the vaccination targets investigated. Their current developmental stage and advantages and disadvantages are highlighted.

Technique	Devices	Vaccine Target	Development Stage	Advantages/Disadvantages
Needle adapter	ID Adapter (West Pharmaceutical Services, Inc., USA)	Poliovirus	Commercially available	**Advantages:** Minimises changes to current clinical practice Low costs **Disadvantages:** Does little to mitigate against fear of needles Does not prevent needle-stick injuries
Microinjection	BD Soluvia™ Micro Injection System (Becton Dickinson, USA) MicronJet600 device (NanoPass Technologies Ltd., Israel) VAX-ID^®^ (Novosanis, Belgium) Immucise (Terumo corporation, Tokyo, Japan)	Hepatitis B Influenza	FDA and CE approved FDA and CE approved Process of obtaining CE approval	**Advantages:** Minimises changes to current clinical practice **Disadvantages:** Separate device needs to be acquired for each injection Does not prevent needle-related injuries
Microneedles	0	Hepatitis B Hepatitis C Influenza Poliovirus SARS-Cov-2 Shigellosis	Phase 1 clinical trials	**Advantages:** Pain-free Allows for self-administration No sharps waste **Disadvantages:** Increased pruritus and erythema rates
Tattooing	Multiple needle tattoo device or permanent make-up device	HPV HIV Lyme disease Melioidosis Tuberculosis	Phase 1 clinical trials	**Advantages:** Utilises commercially available devices **Disadvantages:** Does little to mitigate against fear of needles or reduce pain Does not prevent needle-stick injuries
Jet and ballistic delivery	PowderJect™ (PowderJect, Oxford, UK, acquired by Pfizer) Biojector 2000 (Bioject, USA) Bioject ZetaJet (Bioject, USA) Injex30 (Injex Equidyne, UK) PharmaJet Stratis (PharmaJet, USA) PharmaJet Tropis (PharmaJet, USA) Trigrid electroporation systems (Ichor medical systems, USA) Actranza™ (DAICEL Corporation, Japan)	Dengue Diphtheria, tetanus and pertussis Hepatitis B HPV Influenza Measles, Mumps and Rubella Poliovirus SARS-CoV-2 Rift Valley Fever virus	FDA approved FDA approved FDA approved FDA approved FDA and CE approved CE approved Clinical trials Preclinical	**Advantages:** No sharps waste Effective for DNA vaccines Quick, suitable for mass vaccination **Disadvantages:** Painful Costly to use
Transfollicular	0	Influenza	Clinical/preclinical trials	**Advantages:** Does not disrupt the stratum corneum No sharps waste **Disadvantages:** Tape stripping is painful and time consuming Dependent on diffusion
Thermal ablation	P.L.E.A.S.E.^®^ (Precise Laser Epidermal System) (Pantec Biosolutions, Liechtenstein) UltraPulse^®^ Fractional CO_2_ Laser (Lumenis Inc., UK)	Influenza [188]	Clinical trial CE approved, preclinical tests for vaccines	**Advantages:** No sharps waste Ease of use **Disadvantages:** Two-step process Increased risk of infection
Chemical enhancer	0	Diphtheria Tetanus	Preclinical trials	**Advantages:** No sharps waste Ease of use **Disadvantages:** Frequent side effects Increased risk of infection Dependent on diffusion
Abrasion	STAR Particles Microdermabrasion devices, e.g., MegaPeel^®^ Gold Series (DermaMed International, Lenni, PA, USA)	HIV Tetanus Vaccinia	Preclinical studies FDA approved, preclinical studies for vaccines	**Advantages:** No sharps waste Ease of use Can use existing devices **Disadvantages:** Increased risk of infection Likely not pain-free Dependent on diffusion
Electroporation	CELLECTRA^®^ (Inovio, USA) Easy Vax™ delivery system (Cellectis Therapeutics, Paris, France) Medpulser™ DDS (Inovio, USA)	Dengue Lassa Virus Hepatitis C SARS-CoV-2	Clinical trials Clinical trials CE approved	**Advantages:** Excellent at DNA vaccine transfection **Disadvantages:** Requires additional device Painful
Iontophoresis	ActivaPatch^®^ (North Coast Medical, Inc., USA) Iontopatch^®^ (IontoPatch, USA)	Cancer in preclinical studies	FDA approved FDA approved (neither are applied to vaccines)	**Advantages:** Pain-free Allows for self-administration No sharps waste **Disadvantages:** Patch needs to be worn for extended periods
Ultrasound	0	0	Preclinical trials	**Advantages:** Possibly pain-free Intrinsic adjuvant Suitable for DNA vaccines No sharps waste **Disadvantages:** Requires novel device May be time intensive
